# Supplementary material for: Structure-Aware Mycobacterium tuberculosis Functional Annotation Uncloaks Resistance, Metabolic, and Virulence Genes
Source: mSystems. 2021 Nov 2;6(6):e00673-21. doi: 10.1128/mSystems.00673-21 (PMC8562490; doi:10.1128/mSystems.00673-21)
Supplement: TEXT S1 [file msystems.00673-21-s0001.docx]

**Supplementary text**

**Structure-aware annotation uncloaks *M. tuberculosis* resistance, virulence, and metabolic genes**

^1^Samuel J Modlin, ^1^Afif Elghraoui, ^1^Deepika Gunasekaran, ^1^Alyssa M Zlotnicki, ^2^Nicholas A Dillon, ^1^Nermeeta Dhillon, ^1^Norman Kuo, ^1^Cassidy Robinhold, ^1^Carmela K Chan, ^2^Anthony D Baughn, ^1^Faramarz Valafar*

**^1^**Laboratory for Pathogenesis of Clinical Drug Resistance and Persistence, San Diego State University, San Diego, CA 92182

**^2^**Department of Microbiology and Immunology, University of Minnesota Medical School, Minneapolis, MN, 55455, USA

* Corresponding Author

Content:

[**Manual curation protocol** 3](#_Toc74567697)

[Systematic manual literature curation 3](#_Toc74567698)

[Guidelines for manual product annotation. 3](#_Toc74567699)

[Manual curation quality assurance. 4](#_Toc74567700)

[Enzyme Commission number assignment. 4](#_Toc74567701)

[**Structural Inference protocols** 5](#_Toc74567702)

[Approach 5](#_Toc74567703)

[Precision-informed inclusion criteria 5](#_Toc74567704)

[Hierarchical approach 5](#_Toc74567705)

[Inclusion criteria 6](#_Toc74567706)

[Benchmarking Precision for EC Numbers and GO terms 6](#_Toc74567707)

[Adjusting TM-score to account for model error 9](#_Toc74567708)

[Enzyme commission numbers. 10](#_Toc74567709)

[Gene Ontology Terms. 10](#_Toc74567710)

[Name-based product annotation from structurally similar PDB templates. 10](#_Toc74567711)

[Structure-based transport protein annotation. 11](#_Toc74567712)

[Ligand-binding sites. 12](#_Toc74567713)

[CATH Topology 12](#_Toc74567714)

[**Supplemental Discussion** 12](#_Toc74567715)

[Host mimicry 12](#_Toc74567716)

[Approach & Scope 13](#_Toc74567717)

[Evaluation of previous *in silico* annotation efforts for *M. tuberculosis*. 13](#_Toc74567718)

[Rationale for inclusion criteria and related procedures 13](#_Toc74567719)

[Limitations & strengths 13](#_Toc74567720)

[Manual curation 13](#_Toc74567721)

[Interpreting computationally derived annotations 14](#_Toc74567722)

[Metabolic annotation: enzyme commission number. 14](#_Toc74567723)

[Gene Ontology Terms. 15](#_Toc74567724)

[CATH topologies. 16](#_Toc74567725)

[Ligand-binding sites. 16](#_Toc74567726)

[Resources for further characterization 16](#_Toc74567727)

[Application of approach to other genomes 16](#_Toc74567728)

[**Supplementary Results** 17](#_Toc74567729)

[Comparison of manual annotations to existing databases 17](#_Toc74567730)

[Remaining unannotated genes 17](#_Toc74567731)

[Highlights from structure-derived annotations 17](#_Toc74567732)

[Speculation on function of putative MDMPI homologs 17](#_Toc74567733)

[Distribution of similarity metrics among structure-based annotations 18](#_Toc74567734)

[CATH topologies 18](#_Toc74567735)

[GO terms 18](#_Toc74567736)

[**References** 19](#_Toc74567737)

# **Manual curation protocol**

## Systematic manual literature curation

We created and followed a standardized procedure to systematically curate gene function annotations from the literature (Fig. S1). The annotation of each under-annotated gene was extracted from Mycobrowser (<https://mycobrowser.epfl.ch/> -- formerly known as TubercuList) (1). The set of under-annotated genes comprised 1,057 unannotated (“hypothetical” or “unknown” functional categories) and 668 with ambiguous annotations, totaling 1,725 under-annotated genes. These annotations were converted to feature table format(2) for systematic analysis and editing by curators. Version control through git was used to allow curators to work in parallel and track annotation progress of each gene. Each gene was assigned to two curators to mitigate the risk of missing existing literature annotations, and to reduce the role of human bias in annotation. Each gene was searched in Google scholar in format “mycobac* <gene locus tag>” (e.g. “mycobac* Rv0004”) and confined to publications between January 1st, 2010 and June 30, 2017. Earlier work was included when referenced in publications from the primary search. Patents and non-English articles were excluded. For each publication returned by the query, every mention of the locus tag or gene name was inspected manually unless it became apparent that the article did not contain information relevant to gene product function (e.g., purely an association study). Orthology and domain-based computational annotations were excluded as the sole basis of evidence, since TrEMBL annotations are regularly updated and are quality-controlled, and would likely catch such cases(3). In contrast, orthology and domain-based annotation were included when combined with other evidence, such as using domain annotations to identify candidate genes for subsequent molecular docking simulations that putatively demonstrated product function. Curators evaluated experimental evidence for functional characterization and noted the methodology used to connect gene product and function according to NCBI Feature Table Format(2), using as descriptive language as possible while remaining concise and accurately representing the methods used and conclusions drawn by the primary authors.

## Guidelines for manual product annotation.

To facilitate consistent annotation, we described products in one of three ways:

1. ***high-confidence*** - derived from evidence that, barring human error or data fabrication, definitively prove the annotated function is carried out by the gene product. This highest confidence assignment is implied by the absence of a qualifying adjective. Techniques warranting this annotation include protein purification with subsequent functional characterization through enzymatic assays, and gene knockout/complementation studies that isolate the gene of interest as the causal mechanism behind the observed loss of function.

2. ***probable*** - used for experiments that provide strong evidence that the gene of interest carries out a certain function, but require minor assumptions or rely on strong but fallible inferences. Such techniques include X-ray crystallography with molecular docking and transposon mutagenesis studies.

3. ***putative***- used for experiments where a non-trivial assumption or inference with well-known exceptions is required. Examples include gene-knockout and complementation studies where an observed phenotypic change (e.g., localization of substrate to inner cell-membrane) correlates with KO/complementation status, indicating mediation by the gene of interest. The putative qualifier was also used for *in silico* functional predictions based on three-dimensional methods, such as molecular docking simulations (e.g. PatchDock(4)).

Experiments with insufficient evidence to assign “putative” annotation or higher were left with their product field unchanged, but annotated with a note, using either “potential” or “possible” as qualifiers, where the former connotes relatively higher confidence. The methods justifying these notes require significant assumptions, or are derived from incomplete information, and should be treated as tentative. Notes were also included for well-justified functional information not useful for defining a product name, such as “overexpression increases susceptibility to isoniazid *in vitro*”.

## Manual curation quality assurance.

Each gene query was assigned to two investigators, while a third investigator was designated as "polisher" and served to assure quality and consistency of annotations recorded by the two primary investigators. To hedge against human error, the two investigators curated annotations for each gene, independently. To resolve discrepant annotations, the two investigators would consult with one another to produce a consensus annotation. If two investigators could not resolve the annotation, a third investigator (not necessarily the investigator serving as “polisher”) would act as an arbitrator and break the deadlock if no consensus could be reached.

After manual curation, every gene for which a new function had been assigned was inspected by the polisher. Polishers went to the source from which the new annotation was derived and verified that the conclusions drawn by the initial manual curation were valid, correctly cited, and properly formatted. When the polisher felt the original annotation was inconsistent with annotation guidelines, they and the original curator(s) would discuss discrepant interpretations and form a mutual consensus. If they could not, an additional arbitrator would confer with them to break the deadlock.

## Enzyme Commission number assignment.

Enzyme Commission (EC) numbers are period-delimited, hierarchical descriptors of enzyme-catalyzed reactions. The four EC number digits correspond to progressively granular description of reactions, and when assigned to a protein, imply that it catalyzes the described reaction(5). We annotated all literature-curated gene products with experimentally verified enzymatic activity with EC numbers. We used the EC number assigned by the authors of the source article of the annotation unless it did not follow International Union of Biochemistry and Molecular Biology (IUBMB) conventions(5). When the authors assigned no EC number we assigned one ourselves according the reaction/EC number relations on the official IUBMB database(5). EC numbers were assigned only to the degree of specificity warranted by experimental evidence (e.g. 3.1.-.- when esterase activity was shown, but evidence of no further substrate specificity was provided).

# **Structural Inference protocols**

## Approach

Thresholds informed by EC number precision are approximate and conservative. We included both EC number and GO terms according to these thresholds because both depend on how shared structure implies shared function. They have distinct and complementary advantages: EC numbers comprise a structured hierarchy, allowing thresholds to be set for each tier of specificity. GO terms describes gene products in a species-independent manner, more granularly than EC numbers, and in terms of cellular location, biological process, and molecular function(6, 7), making them useful for relating gene products across diverse databases and inferring non-metabolic functions.

### Precision-informed inclusion criteria

We designed our inclusion criteria based on how likely an annotation would reflect true function, formalized mathematically as precision (Equation 1, main text). We used published precision benchmarks(8, 9) where applicable, and emulated their benchmarking procedure where not using *M. tuberculosis* sample data to create our own. We had applicable precision benchmarks for LBS, CATH, and general structure conservation based on and Template-modelling score (“TM-score”, a measure of structural similarity independent of protein length(10)), as well as how true TM-score deviates as a function of model quality, (C-score), but lacked adequate benchmarks for incorporating GO terms and EC numbers. We had intended to use recently published benchmarks for predictions from the newest version of COFACTOR(11). However, GO terms are predicted using a different algorithm in the new version, which is not part of I-TASSER standalone (v5.1). Algorithms for inferring EC and LBS, however, remain the same between these versions of COFACTOR (personal communication). We used these benchmarks for LBS incorporation but used our own as determined below for EC numbers (and extended them to GO terms).

### Hierarchical approach

To determine final gene product annotations, we prioritized more reliable methods of inference before deferring to less reliable methods. We first included manual annotations (highest priority) and high-confidence EC-based annotations from structural similarity. EC-based annotations were considered with manual annotations so that secondary functions of moonlighting proteins would not be precluded from annotation when manual curations were present. EC annotations were included when projected precision exceeded 50% (Figs. S2D and S3A), while GO terms were incorporated hierarchically (Fig. S3B).

Rather than directly mapping PDB template name to product annotations, we derived product names using EC number and GO terms (Fig. S3). This enabled transfer of shared aspects of similar proteins without implying identical function. For example, a protein structure model may be similar enough to confidently transfer “methyltransferase” as an annotation, but not “6-methyladenine DNA methyltransferase”, the macromolecule name associated with the PDB template of similar structure. Using the name of exact PDB match would imply greater specificity than the degree of structural similarity warrants, which would be misleading in some cases.

Manual annotations, EC numbers, and GO terms were annotated in a common hierarchy because they describe general function and thus less frequently have an additional distinct function (though possible, hence the inclusion of multiple EC numbers in some cases, Fig. S3). In contrast, CATH topologies annotate structural fold alone(12), which though highly correlated to function, is orthogonal to GO and EC annotations. Ligand-binding site (LBS) annotations are local protein features, and are thus also an orthogonal attribute to overall product assignment. Therefore, LBS and CATH topologies were annotated irrespective of other annotations.

## Inclusion criteria

Amino acid (AA) sequences were extracted from TubercuList and run through a local installation of I-TASSER for structural prediction and identification of candidate homologs and analogs in the protein data bank (PDB) through structural alignment(8, 9). From I-TASSER, metrics are computed that describe similarity between the modeled structures and solved structures on PDB. For each structure model/PDB template protein pair, similarity metrics were extracted along with EC, GO, and CATH annotations of the known protein (from PDB(13)). One could argue that the COFACTOR sequence-structure-function benchmarks recommended by I-TASSER are adequate since they have been trained extensively to derive function based on global and local similarity(14). However, the most recent version COFACTOR (which uses a new algorithm for GO term annotations) was not incorporated into the standalone version of I-TASSER, it was not amenable to the high-throughput analysis employed in this study. Our rationale is explained further in subsequent sections.

### Benchmarking Precision for EC Numbers and GO terms

To estimate precision (Equation 1, main text) from the confidence “C-scoreGO”, and “C-scoreEC”, not to be confused with “C-score_I-TASS_“ used by I-TASSER structural prediction (see Glossary for clarification of terms) calculated by COFACTOR(11, 15) of the I-TASSER suite(14, 16), We ran sample AA sequences of known function through I-TASSER (Iterative Threading ASSEmbly Refinement) suite (standalone, version 5.1) to observe how similarity metrics (TM-score, AA%, and C-score) correlated with precision. We then assessed how precision of EC number and GO term predictions correlated with similarity metrics through logistic regression (Fig. S2) to identify those metrics most predictive of precision. We used 50% and 75% as the cutoff for usage of “putative” and “probable” adjectives, respectively. We focused two metrics that best correlated with precision (Fig. S2D): AA identity (AA%) and TM-score).

TM-score describes structural similarity ranging from 0 and 1. It represents the average root-mean squared deviation across all atoms in the structural prediction with respect to the PDB template, normalized to remove apparent deviation arising falsely due to local differences(9, 10, 17) (Equation 1, as calculated by Zhang and Skolnick(10)).

$\text{TM-score}=\left( 1/L_{N} \right)\sum_{i=1}^{L_{T}} \frac{1}{\left( 1+d_{i}^{2}+d_{0}^{2} \right)}$ (1)

where *L_N_* is protein length, *L_T_* is the length of the aligned residues to the template, *d_i_* is the distance of the *i*^th^ pair of residues between two structures after an optimal superposition, and $d_{0}=1.24\sqrt[3]{L_{N}-15}-1.8$, as described by Zhang *et al.*, normalizes for protein length, thereby allowing structural similarity to be compared across proteins of different length(10).

We derived our sample data from under-annotated gene functional annotations manually curated with “probable” confidence or higher (which totaled 163 genes, Fig. S2D, Dataset 1) and 200 characterized genes chosen at random. EC numbers were extracted from these 363 genes and GO terms marked as experimentally verified in UniProt(3). We ran these 363 sequences through I-TASSER to benchmark precision as a function of several similarity metrics. We used a binomial logistic regression on these sample data to relate precision (Equation 1, main text) of EC and GO predictions to C-scores and TM-scores generated by I-TASSER. The regressions used maximum likelihood estimates for the generalized linear model using iteratively re-weighted least squares with a logit linking function, implemented in R. From these regressions we approximated the probability of two protein structures sharing functions as encoded by EC or GO annotations as a function of similarity metrics (Fig. S2).

To determine which metrics could best inform inclusion protocols, we required any relation of similarity metric to precision to satisfy two criteria:

1) Precision must increase monotonically as a function of the similarity metric.

2) For hierarchical frameworks (e.g., EC numbers), less specific tiers must have greater precision at any given x-value along their regression lines. Violation of this criterion would interfere with boundaries between hierarchy levels.

In addition to AA% and TM-score, functional predictions from COFACTOR are generated and are recommended for inferring function from model structure(11). However, we found C-scoreEC (a confidence metric from COFACTOR) unfit for this use on our data: while precision increased monotonically with both C-scoreEC and µGEOM across all EC levels of the regression, the COFACTOR C-scoreEC second EC-digit regression line crossed that of the first EC digit (Fig. S2C), violating our second criterion. Furthermore, the set of EC:PDB mappings utilized by COFACTOR had far fewer hits of any value than that directly from PDB annotations (can be seen from the relative abundance of dark translucent circles in Fig. S2C versus Fig. S2D). This relatively sparse representation of COFACTOR EC:PDB mapping likely owes to the limited library of EC number:structure relations in the BioLIP database utilized by COFACTOR(11, 15). These observations lead us to choose µ_geom_ as a more useful metric for estimating model precision from I-TASSER output for our purposes.

Though precision for GO annotations from all three ontologies increased (Fig. S2A) monotonically with C-scoreGO, precision had a low ceiling, likely owing to the varying degree of completeness in GO annotations, a consequence of their redundancy^8^. In contrast to the regression results in Fig. S2A and Fig. S2C, EC number precision as a function of µ_geom_ was higher at all levels of similarity metrics, and inclusion thresholds were thus determined using precision of EC annotations according to µ_geom_ (Fig. S2D).

After seeing that both C-scoreEC and C-scoreGO violated our criteria for a suitable metric, we surveyed how matching and discordant EC assignments between modeled protein structures and PDB templates similar to I-TASSER structure predictions distributed with respect to sequence identity (AA%) and structural similarity (TM-score). As expected, the proportion of EC assignments discordant between training gene products and predicted homologs increased with specificity of EC tier evaluated (Fig. S2B). AA% and TM-score correlated strongly with one another (R=0.784, Pearson correlation coefficient), among both concordant and discordant EC numbers (Fig. S2B), suggesting both are informative for setting inclusion thresholds. To represent both AA% and structural similarity, we took their geometric mean (which we call “µ_geom_”).

We regressed precision against µ_geom_ for each EC digit to inform our inclusion criteria based on expected precision (Fig. S2D). We used this estimated precision as the 50% and 75% cutoffs for “putative” and “probable” qualifying adjectives (Fig. 1, main text).

**C**

**D**

### Adjusting TM-score to account for model error

The relationship between precision and TM-score has been rigorously benchmarked in prior work by Xu and colleagues(9). This relation between TM-score and precision for matching CATH topologies (which can be thought of as sharing the same “fold”) between two proteins follows an extreme value distribution(9), which confers high discriminatory power to a binary inclusion threshold. We set the minimum TM-score for inclusion as that which corresponded to 52% precision, after correcting TM-score for the expected modeling error (Equation 3).

TM-score is calculated by comparing predicted protein structure (computed by I-TASSER) and the PDB template structure (which is known). To relate to the true similarity between modeled protein structure and PDB structure, we use the C-score to estimate the degree to which true TM-score differs from the TM-score of the model (“TM-scoremodel🡪native”, Equation 1). Zhang and colleagues trained 300 proteins and found that C-scoreI−TASS correlates with the TM-score between predicted and true structure through the relation(9):

*TM-scoremodel🡪native* = 0.0006 ∗ *C-score*^2^ + 0.13 ∗ *C-score* + 0.71 (2)

where *TM-scoremodel🡪native* is the difference between the predicted and native structure of the gene product of interest. We adjust our TM-score accordingly:

*TM-scoreADJ* = *TM-scorePDB* − 0.83(1 - *TM-scoremodel🡪native*) (3)

which yields the adjusted TM-score (*TM-scoreADJ* or *TM_ADJ_*). *TM-scorePDB* refers to the TM-score between protein of interest model and PDB template (referred to elsewhere as “TM-score”, but renamed to remove ambiguity clarity in Equation 3). We then used this as the threshold based on the posterior probability of proteins sharing CATH topology at a given TM-score(9). The coefficient of 0.83 is used to subtract the degree of difference between the model and the native structure that would be expected to move away from the PDB template (two randomly oriented proteins average a TM-score of 0.17(9)).

### Enzyme commission numbers.

EC number annotations were transferred to the degree of specificity dictated by the threshold they met (Fig. S2D). If no EC-bearing PDB structure matches passed a threshold, the protein structure model progressed to the GO protocol, during which EC numbers were inferred for some products (see next section). Conflicting EC numbers were pruned to a common digit if possible and collapsed into unique ECs at the degree of specificity dictated by their similarity if not. Final EC numbers informed product field annotations. In cases where product field had a prior entry, if the EC annotations were incorporated according to the flow-diagram in Fig. S3A. Threshold boundaries were set at 75% and 50% precision for the 4th and 3rd digits of specificity in EC numbers (Fig. S2D and Fig. 1 of the main text).

### Gene Ontology Terms.

Gene products unannotated by the EC inclusion protocol were screened for PDB matches at progressively lower µ_geom_ thresholds for GO term transfer (Fig. S3B). With this approach, gene products receive only the best available annotations, since those already annotated (from sources of higher expected precision) exit the iteration before progressing to weaker thresholds. Unlike using a single threshold, this approach mitigates over-annotation with assignments of lower confidence, without lowering the number of annotated genes. In the final gff annotation, each GO term transferred was labeled with all the PDB templates supporting it. EC numbers were inferred programmatically and included where a direct mapping between EC and GO term existed. If the GO term implied enzymatic activity, relevant terms were searched in EXPaSY ENZYME(18) and an EC number was assigned to the gene and used to derive the product name from the ENZYME.dat file obtained from the EXPaSY database. When GO terms mapped to multiple EC numbers, the EC numbers were merged at the most specific level at which they converged (e.g., 3.2.1.5 and 3.2.2.4 would resolve to 3.2.-.-). Such instances of EC assignments from GO terms occurred often because of inconsistent annotation across frameworks in PDB entries: many PDB entries were not assigned EC numbers, but were assigned GO terms based on enzymatic activity. If GO terms were transferred but none mapped to EC numbers, GO terms were examined for terms sufficiently descriptive to constitute a product name (e.g., “DNA binding transcription factor activity” is sufficiently descriptive whereas “pathogenesis” is not). All GO terms remain in the annotation file (S1 Annotation), but where no GO term warranted product annotation, the product field was left unchanged, and not included in the final counts (Results, main text).

### Name-based product annotation from structurally similar PDB templates.

Many proteins with quality structure models (C-score > -1.5) had similarity only to PDB templates that lacked EC or GO term annotations. These were algorithmically included as matches using the same µ_geom_ thresholds described for EC and GO inclusion (Fig. S3) but assigned product names manually. The top 3 PDB templates of genes lacking functional annotation (EC, GO, or manual) were examined. Each pairwise relation between modeled protein structure and PDB structure with a TM-score greater than 0.85 and/or µ_geom_ meeting the inclusion criteria for putative EC 3rd digit (geometric mean > 0.374, corresponding to a precision > 0.5; Fig. 1 of main text) were considered for annotation by the following criteria. When both of the criteria below were met, annotation was transferred to the degree of specificity warranted by the geometric mean, and product names were assigned as detailed for GO terms (Fig. S3B):

1. The portion of the modeled protein structurally with the PDB entry crystal structure coincided with the coordinates annotated with the functional motifs responsible for the function under consideration for transfer.
2. The PDB template had the function verified experimentally in its UniProt entry or in the primary publication that claimed to elucidate its function (typically in the same article that determined its structure).

### Structure-based transport protein annotation.

Though µ_geom_ proved useful for estimating precision, the sample distribution of AA% and TM-score did not cover areas where one was high and the other low (Fig. S2B). While high AA% should be captured by annotation methods based on sequence identity, high TM-score with low AA% is only amenable to annotation through structural similarity. However, the relationship between TM-score and precision of functional annotations in such cases is unclear. For these cases with high TM-score, we transferred “CATH structural annotations” from similar PDB entries based on a relationship between precision and TM-score determined previously(9). We also implemented special inclusion criteria for protein classes that typically lack sequence similarity despite sharing structure and function.

Transport proteins require different inclusion criteria than globular proteins. These proteins are especially difficult to characterize experimentally, and more conserved in structure than in sequence, relative to globular proteins(19, 20). To accommodate these unique features, inclusion criteria for transferring annotations from PDB templates of transport proteins weighted structural similarity more heavily than AA%: These annotations were transferred if both of the following were true:

1. Greater than 90% of the PDB entry protein implicated in transport structurally aligned to by the modeled protein structure.
2. Structural similarity exceeded the threshold for CATH topology transfer.

Transport protein annotations were transferred in a less specific form than that given the PDB templates (e.g. “transport protein” instead of “Na+/H+ antiporter”), unless all three highest templates matched a more specific description and the TM-score exceeded 0.85, in which case a more specific product name was transferred.

### Ligand-binding sites.

Ligand-binding sites (LBS) were included from COFACTOR at C-score_LBS_ values corresponding to precision > 0.6 in a recent benchmarking study(11).

### CATH Topology

CATH is a hierarchic classification system of protein domain structures, in which “topology” is the third level of the hierarchy, more specific than “architecture” and more general than “superfamily”(12, 21). Structural fold annotations can be functionally informative in some cases. CATH topologies were annotated according to a previously established posterior probability distribution(9), and included when their precision exceeded 0.52.

# **Supplemental Discussion**

## Host mimicry

Annotation based on structural similarity can identify potential *M. tuberculosis* effector proteins, though care must be taken to carefully vet such candidates, since incidental matches with eukaryotic proteins on PDB is expected. GO terms of the cellular component ontology further implicate host-pathogen interaction and eukaryote-specific organelles immune niches such as “exosome”(Fig. S5D), an access point for pathogen-derived effectors(22)(23)^,^. Proteins annotated with these terms provide potential candidate effector proteins for future investigation, though it is likely than some are merely an effect of PDB being filled primarily with human proteins. Additional potential effectors can be found among the significantly similar structures to under-annotated genes in Dataset 3.

## Approach & Scope

### Evaluation of previous *in silico* annotation efforts for *M. tuberculosis*.

Previous large-scale *in silico* attempts at predicting hypothetical gene function were considered(25–29) for integration, but we did not incorporate their results in this work because they were not assigned clear confidence metrics. We instead opted to run the amino acid sequences for the 1,725 under-annotated genes through I-TASSER(8) and evaluated whether the suite could produce informative *in silico* predictions.

### Rationale for inclusion criteria and related procedures

Manual annotations, EC numbers, and GO terms were annotated hierarchically among one another because they describe general function (except for GO terms describing local properties, e.g. “metal-ion-binding protein”), and thus a gene product correctly annotated with a function is less likely to truly have an additional, distinct function (though possible, hence the inclusion of multiple EC numbers in some cases, Fig. S3A). In contrast, CATH topologies annotate structure alone, which, while ultimately giving rise to function, does not necessarily carry functional meaning. Ligand-binding site (LBS) annotations are orthogonal to both annotation of large structural features and overall gene product function, as they are dictated primarily by local structure. Therefore, LBS and CATH topologies were annotated irrespective of EC, GO, or manual annotation, as well as one another.

We used few (“putative” and “probable”) broad classifications rather than many granular ones to:

1. Minimize false prioritization of PDB templates that appear more similar to native protein structure than true matches, due to inherent variability in structure prediction
2. Identify instances where templates with a similar degree structural similarity differed in function, for careful assessment.

By iteratively including annotations with coarse hierarchical steps, we can differentiate of degrees of similarity when their difference is large (and thus more often encoding true differences in degree of similarity) without errantly detection false differences that appear spuriously due to imperfections in predicting structure.

## Limitations & strengths

### Manual curation

While automated curation software can help curators prioritize manual curation but current software tools developed to “read” scientific articles and extract information cannot reliably gauge strength of determination methods, leaving manual curation as the gold standard(30). Manual curation is a massive task: UniProt estimates their curators evaluated over 4,500 papers(30), and our manual curations drew from 656 and required reading thousands. Programmatic access of PubMed Central (PMC) can reduce the human workload required for literature curation and facilitate up-to-date functional annotation for a particular organism such as *M. tuberculosis*. We recommend the TB community follow UniProt’s approach of 1) regularly reading through published articles to keep literature review feasible and 2) automate filtering through articles to distill those containing evidence meriting functional annotation(30). Standardized criteria and usage of well-structured ontologies can keep annotation accuracy high, though absolute objectivity is challenging. Ontologies for several facets of annotation exist, including evidence assignment, function, and relationships between gene products(31, 32). The volume of new publications is simply too large for global curation efforts like UniProt to manually annotate all experimental characterizations. Research communities of widely studied organisms must initiate their own, complementary manual curation efforts to remain updated due to the sheer volume of publications and the unique features of different organisms. Lastly, some non-ambiguous functions may have been contradicted by recent work. We did not attempt to resolve such conflicts and focused only on the 1,725 classified as underannotated.

### Interpreting computationally derived annotations

To complement our manual literature curation, we further annotated the 1,725 under-annotated genes through inferential methods. Each of these annotation sources have their own set of advantages, assumptions, limitations, discussed below. Error and bias in resolving protein structure and function are numerous, incompletely characterized, and distributed unevenly across protein classes and families(33). This uncertainty and heterogeneity make accounting explicitly for sources of error challenging and time-consuming, if not intractable. To circumvent complications in accounting for these errors explicitly, we assessed how the geometric mean of amino acid similarity and structural similarity related to precision of controls. This approach to captures PDB annotation reliability in a single metric and informed our inclusion criteria. With this approach, precision is considered independently of how PDB entries were annotated, and precision reflects what was attained despite potential flaws or inconsistencies in PDB annotation procedures. Future work could develop more nuanced methods to account for heterogeneity of error frequency more explicitly, or contingent on a greater number of variables.

### Metabolic annotation: enzyme commission number.

The binomial regression in Fig. S2D does not definitively predict precision at a given value of µ_geom_, nor was it designed to. Rather, it provides a clear interpretation of how terms were derived, and an approximate, conservative projection of precision; This projected precision likely underestimates true precision: False negatives occur when the query protein EC is predicted and one of the following occurs:

1) The EC of the PDB template is incorrectly annotated.

2) The PDB template is incompletely annotated (the reaction described by the EC has multiple valid EC assignments).

3) The protein of the PDB template has multiple true ECs, of which only a subset are annotated.

4) Our EC assignment was incomplete or incorrect for reasons 1-3.

In contrast, false positives are vanishingly rare; either the query or PDB EC assignment would have to be incorrect on a particular EC number of over 7,000(5).

Annotations were incorporated hierarchically using boundaries of 50% and 75% projected precision for the 3rd and 4th EC tiers (Fig. 1 of main text). The 3rd level of EC numbers describes detailed catalytic function (e.g., 3.1.6.- describe “Sulfuric ester hydrolases”), only lacking their specific preferred substrate(s).

### Gene Ontology Terms.

The GO framework is unique in that it describes gene products in a species-independent manner and at varying degrees of specificity(32, 34). These features make GO terms useful for relating gene products across databases and drawing parallels between products from different species share function not apparent in their primary names. For example, "multidrug resistance protein" and "neurexin 1" sound unrelated, but can be unified by the GO term "transmembrane transport". This cross-species unification is particularly useful for reconciling annotation transfers of analogs and distant homologs into gene product names relevant to the organism of interest.

GO terms convey meaning at multiple functional levels and should be interpreted differently depending on their ontology and specificity. Molecular function ontology terms are likely the most reliable; they typically convey information that depends less on the genetic background of the organism than the other ontologies and are thus more likely conserved across similar structures in different organisms.

We implemented inclusion criteria for GO terms mirroring EC number cutoffs because they ultimately convey similar information: the propensity of structural similarity to PDB templates to imply shared function. GO terms encode diverse biological information and are frequently used to identify enriched processes and functions in a set of genes, such as those with upregulated expression under a particular condition, or those found mutated across clinical isolates during treatment of a particular drug. These GO terms increase the annotation coverage of *M. tuberculosis* H37Rv reference genome, and may help to uncover latent commonalities among gene sets, making them a useful component of this annotation update.

### CATH topologies.

Topology annotations derived through CATH had limited AA% in most instances (Fig. S5) which makes their incorporation valuable where genes would have no annotation otherwise(35). Closer inspection of topologies with little functional diversity could allow valid inference that the protein of interest shares the function common to the topology, but we did not attempt to make such distinctions in this work. We highlight proteins assigned Tetracycline repressor folds with relative confidence in Dataset 3M, since this fold has a narrow range of functions and many proteins with this fold play important roles in *M. tuberculosis*. The most frequently occurring CATH topologies are displayed in Dataset 3L.

### Ligand-binding sites.

Ligand-binding sites (LBS) were included from COFACTOR at C-score_LBS_ values corresponding to precision > 0.6 in a recent benchmarking study(11). LBS have narrower use than EC or GO terms, but can identify putative ligand-protein interactions, and elemental requirements for gene function. They can also help to elucidate gene function when the ligand is specialized for a particular class of reactions, and can, in some cases, help discriminate within structurally conserved folds of diverse function.

## Resources for further characterization

For accessibility, we provide final annotations in common machine and human readable formats. We provide a machine-readable GFF3 file containing our updated annotation (S1 Annotation). This file includes EC numbers, GO terms, and product name annotations, and can be used for reference transfer, variant effect prediction, and other bioinformatic analyses.

Annotations in the GFF3 are included according to our inclusion criteria. PDB templates with structures similar yet below our criteria can consult Dataset 3B, which contains the top 3 PDB templates for each of the 1,711 under-annotated genes based on µ_geom_, and Dataset 3A, which holds all matches where a TM_ADJ_ > 0.52 (Equation 3) and/or µ_geom_ > EC3 (putative) threshold. Further exploration of modeled protein structures can be carried out by analyzing the I-TASSER output for each of the under-annotated genes at <https://tuberculosis.sdsu.edu/structures/>. These include functional predictions by COFACTOR, predicted ligand binding sites, local secondary structure confidence, and other potentially useful metrics.

## Application of approach to other genomes

Our hierarchical, precision-guided approach to incorporating annotations protects against overannotation while increasing annotation coverage. These are desirable features for expanding annotation of any organism and should become more effective over time: Structural prediction methods should improve in accord with algorithm design, become more accessible as computational costs lower, and provide greater coverage as more protein structures and functions are elucidated. While we implemented this approach to maximize annotation of *M. tuberculosis*, it can be applied to other species. Employing these approaches to other species would help reconcile functional characterizations in the literature with what can be inferred from conservation of structure and function and increase quality of functional data.

# **Supplementary Results**

## Comparison of manual annotations to existing databases

We compared the 282 products with manually curated annotations from literature to four other frequently cited databases. We initially compared our manual annotations to UniProt’s because their team of curators follow a standardized manual protein annotation curation protocol, and regularly update annotations(30) (Fig. S4A). This comparison revealed 139 genes with manual annotations absent in UniProt and an additional 33 genes with manual curations more complete than in UniProt (Fig. S4A). We then compared these 172 genes to Entrez, Mtb Network Portal, and PATRIC, revealing 118 genes more thoroughly annotated in our manual curation than in any of the databases, and 54 not solely annotated as an antigen (Fig. S4A). In total, 135 previously under-anntoated received some level of new annotation (68 not solely as antigens). We distinguished genes annotated solely as antigens from those with other annotations because while antigenic properties are the primary function of some proteins, many have different primary functions and are simply recognized by the host.

## Remaining unannotated genes

## Highlights from structure-derived annotations

### Speculation on function of putative MDMPI homologs

Considering that DinB-like enzymes are more numerous in species with a large capacity for secondary metabolite synthesis, like *Mycobacteria*(42) (e.g., polyketide & nonribosomal peptide synthesis genes), detoxifying secondary metabolite byproducts presents a rational speculative function. Elaborating on the genomic context and experimental data used to speculate potential function for the four DinB-like enzymes in Fig. 4H, Rv0036 shares an operonic configuration analogous to *Bacillus subtilis* DinB family bacillothiol-dependent S-transferase YfiT, with a downstream MFS family transporter, consistent with a function in detoxification. Comparative proteomics has shown Rv0036c is more abundant in intraphagosomally-grown multidrug-resistant *M. tuberculosis* than in broth culture(43). Meanwhile, Rv0738 is more abundant in biofilm (a highly hypoxic environment) than planktonic bacilli(44), and Rv2036 is co-operonic with an ArsR repressor protein (Rv2034) that upregulates master regulators of hypoxic response (DosR)(45) and virulence (PhoP)(46). Rv2036 expression is strongly correlated Rv2034 (<http://galaganlab.bu.edu/tbdb_sysbio/CC/Rv2036.html>), suggesting related function. Rv1727, its upstream gene Rv1726, and Rv1872c—putative L-Lactate dehydrogenase implicated in iron acquisition through sequestering transferrin(47)—form a gene hub(48) differentially transcribed between two closely related (yet phenotypically distinct) clinical isolates under carbon limitation. This observation is consistent with the predicted iron-coordinating site of Rv1727, suggesting it may be involved in responding to iron limitation stress response.

### Distribution of similarity metrics among structure-based annotations

We classified PDB template hits into candidate annotation categories according to the regression of precision against TM-score and AA%. More PDB templates qualified for transfer of lower confidence and specificity thresholds than for higher tiers (Fig. S5B). Many templates with high TM-score but low AA% qualified for CATH annotation transfer, which underscores the utility of structure-based annotation in the absence of sequence homology.

Several PDB templates were 100% identical to query proteins, representing protein sequences of *M. tuberculosis* or closely related mycobacteria (Fig. S5B). Overall, model quality was high for annotations that passed inclusion criteria; PDB:query relationships meeting criteria for EC, GO or CATH inclusion had a mean C-score of 0.634 and distributed according to their confidence and specificity (Fig. S5A). Similarly, model quality of relations not meeting inclusion criteria (gray and black) were lower than the relations meeting any of the inclusion criteria (red, greens, and blues, Fig. S5B), demonstrating annotations were derived from high quality structural models, rather than false similarity from noisy structural predictions.

### CATH topologies

Transcriptional repressors dissociate from their target DNA in the presence of substrate, activating transcription, which, in some cases, are antibiotics, or other compounds of clinical interest(49). Genes that met criteria for annotation with the “Tetracycline Repressor; domain 2” topology are in Dataset 3M.

### GO terms

GO terms describe gene products through three structured ontologies: biological processes (the processes in which the product plays a part), cellular components (its location within the cell), and molecular functions (its specific function)(6, 7). The distribution of GO terms transferred to under-annotated genes because of structural similarity indicates common themes in the new annotations, (Fig. S5D). Potentially of interest are common GO Cellular component GO terms include host organelles (e.g. “nucleus” and “mitochondrion”) and at the host-pathogen interface (e.g. the 27 genes annotated with “extracellular exosome”), possibly indicating candidate host-interaction proteins.

# **References**

1. Lew JM, Kapopoulou A, Jones LM, Cole ST. 2011. TubercuList – 10 years after. Tuberculosis 91:1–7.

2. Detailed Annotation Guide.

3. UniProt Consortium. 2017. UniProt: the universal protein knowledgebase. Nucleic Acids Res 45:D158–D169.

4. Sharma P, Kumar B, Gupta Y, Singhal N, Katoch VM, Venkatesan K, Bisht D. 2010. Proteomic analysis of streptomycin resistant and sensitive clinical isolates of Mycobacterium tuberculosis. Proteome Sci 8:59.

5. Webb EC, others. 1992. Enzyme nomenclature 1992. Recommendations of the Nomenclature Committee of the International Union of Biochemistry and Molecular Biology on the Nomenclature and Classification of Enzymes. Academic Press.

6. Ashburner M, Ball CA, Blake JA, Botstein D, Butler H, Cherry JM, Davis AP, Dolinski K, Dwight SS, Eppig JT, Harris MA, Hill DP, Issel-Tarver L, Kasarskis A, Lewis S, Matese JC, Richardson JE, Ringwald M, Rubin GM, Sherlock G. 2000. Gene ontology: tool for the unification of biology. The Gene Ontology Consortium. Nat Genet 25:25–29.

7. Holliday GL, Davidson R, Akiva E, Babbitt PC. 2017. Evaluating Functional Annotations of Enzymes Using the Gene Ontology, p. 111–132. *In* The Gene Ontology Handbook. Humana Press, New York, NY.

8. Yang J, Zhang Y. 2015. Protein Structure and Function Prediction Using I-TASSER. Curr Protoc Bioinforma 52:5.8.1-15.

9. Xu J, Zhang Y. 2010. How significant is a protein structure similarity with TM-score = 0.5? Bioinformatics 26:889–895.

10. Zhang Y, Skolnick J. 2004. Scoring function for automated assessment of protein structure template quality. Proteins Struct Funct Bioinforma 57:702–710.

11. Zhang C, Freddolino PL, Zhang Y. 2017. COFACTOR: improved protein function prediction by combining structure, sequence and protein–protein interaction information. Nucleic Acids Res 45:W291–W299.

12. Dawson NL, Lewis TE, Das S, Lees JG, Lee D, Ashford P, Orengo CA, Sillitoe I. 2017. CATH: an expanded resource to predict protein function through structure and sequence. Nucleic Acids Res 45.

13. Rose PW, Prlić A, Altunkaya A, Bi C, Bradley AR, Christie CH, Costanzo L Di, Duarte JM, Dutta S, Feng Z, Green RK, Goodsell DS, Hudson B, Kalro T, Lowe R, Peisach E, Randle C, Rose AS, Shao C, Tao Y-P, Valasatava Y, Voigt M, Westbrook JD, Woo J, Yang H, Young JY, Zardecki C, Berman HM, Burley SK. 2017. The RCSB protein data bank: integrative view of protein, gene and 3D structural information. Nucleic Acids Res 45:D271–D281.

14. Yang J, Yan R, Roy A, Xu D, Poisson J, Zhang Y. 2015. The I-TASSER Suite: protein structure and function prediction. Nat Methods 12:7–8.

15. Roy A, Yang J, Zhang Y. 2012. COFACTOR: an accurate comparative algorithm for structure-based protein function annotation. Nucleic Acids Res 40:W471–W477.

16. Roy A, Kucukural A, Zhang Y. 2010. I-TASSER: a unified platform for automated protein structure and function prediction. Nat Protoc 5:725–38.

17. Zhang Y, Skolnick J. 2005. TM-align: a protein structure alignment algorithm based on the TM-score. Nucleic Acids Res 33:2302–9.

18. Bairoch A. 1994. The ENZYME data bank. Nucleic Acids Res 22:3626–3627.

19. Theobald DL, Miller C. 2010. Membrane transport proteins: surprises in structural sameness. Nat Struct Mol Biol 17:2–3.

20. Olivella M, Gonzalez A, Pardo L, Deupi X. 2013. Relation between sequence and structure in membrane proteins. Bioinformatics 29:1589–1592.

21. Orengo CA, Michie AD, Jones S, Jones DT, Swindells MB, Thornton JM. 1997. CATH – a hierarchic classification of protein domain structures. Structure 5:1093–1109.

22. Schorey JS, Cheng Y, Singh PP, Smith VL. 2015. Exosomes and other extracellular vesicles in host–pathogen interactions. EMBO Rep 16:24–43.

23. Smith VL, Cheng Y, Bryant BR, Schorey JS. 2017. Exosomes function in antigen presentation during an in vivo Mycobacterium tuberculosis infection. Sci Rep 7.

24. Kolli N, Garman SC. 2014. Proteolytic Activation of Human Cathepsin A. J Biol Chem 289:11592–11600.

25. Ramakrishnan G, Ochoa-Montaño B, Raghavender US, Mudgal R, Joshi AG, Chandra NR, Sowdhamini R, Blundell TL, Srinivasan N. 2015. Enriching the annotation of {Mycobacterium} tuberculosis {H}37Rv proteome using remote homology detection approaches: {Insights} into structure and function. Tuberculosis 95:14–25.

26. Mao C, Shukla M, Larrouy-Maumus G, Dix FL, Kelley LA, Sternberg MJ, Sobral BW, de Carvalho LPS. 2013. Functional assignment of Mycobacterium tuberculosis proteome revealed by genome-scale fold-recognition. Tuberculosis. Churchill Livingstone.

27. Al-Khafaji ZM. 2013. In Silico Investigation of Rv Hypothetical Proteins of Virulent Strain Mycobacterium tuberculosis H37Rv. ndian J Pharm Biol Res 1:81–88.

28. Doerks T, Noort V van, Minguez P, Bork P. 2012. Annotation of the M. tuberculosis Hypothetical Orfeome: Adding Functional Information to More than Half of the Uncharacterized Proteins. PLoS One 7:e34302.

29. Mazandu GK, Mulder NJ. 2012. Using the underlying biological organization of the Mycobacterium tuberculosis functional network for protein function prediction. Infect Genet Evol 12:922–932.

30. Poux S, Arighi CN, Magrane M, Bateman A, Wei C-H, Lu Z, Boutet E, Bye-A-Jee H, Famiglietti ML, Roechert B. On expert curation and scalability: UniProtKB/ Swiss-Prot as a case study.

31. Ashburner M, Ball CA, Blake JA, Botstein D, Butler H, Cherry JM, Davis AP, Dolinski K, Dwight SS, Eppig JT, Harris MA, Hill DP, Issel-Tarver L, Kasarskis A, Lewis S, Matese JC, Richardson JE, Ringwald M, Rubin GM, Sherlock G. 2000. Gene Ontology: tool for the unification of biology. Nat Genet 25:25–29.

32. The Gene Ontology Consortium, Gene Ontology Consortium, The Gene Ontology Consortium. 2017. Expansion of the Gene Ontology knowledgebase and resources. Nucleic Acids Res 45:D331–D338.

33. Schnoes AM, Brown SD, Dodevski I, Babbitt PC. 2009. Annotation Error in Public Databases: Misannotation of Molecular Function in Enzyme Superfamilies. PLoS Comput Biol 5.

34. Hill DP, Davis AP, Richardson JE, Corradi JP, Ringwald M, Eppig JT, Blake JA. 2001. PROGRAM DESCRIPTION: Strategies for Biological Annotation of Mammalian Systems: Implementing Gene Ontologies in Mouse Genome Informatics. Genomics 74:121–128.

35. Zhang Y. 2014. Interplay of I-TASSER and QUARK for template-based and ab initio protein structure prediction in CASP10. Proteins 82 Suppl 2:175–187.

36. Ma S, Minch KJ, Rustad TR, Hobbs S, Zhou S-L, Sherman DR, Price ND. 2015. Integrated Modeling of Gene Regulatory and Metabolic Networks in Mycobacterium tuberculosis. PLoS Comput Biol 11:e1004543.

37. Reddy TBK, Riley R, Wymore F, Montgomery P, DeCaprio D, Engels R, Gellesch M, Hubble J, Jen D, Jin H, Koehrsen M, Larson L, Mao M, Nitzberg M, Sisk P, Stolte C, Weiner B, White J, Zachariah ZK, Sherlock G, Galagan JE, Ball CA, Schoolnik GK. 2009. TB database: an integrated platform for tuberculosis research. Nucleic Acids Res 37:D499–D508.

38. Gillespie JJ, Wattam AR, Cammer SA, Gabbard JL, Shukla MP, Dalay O, Driscoll T, Hix D, Mane SP, Mao C, Nordberg EK, Scott M, Schulman JR, Snyder EE, Sullivan DE, Wang C, Warren A, Williams KP, Xue T, Seung Yoo H, Zhang C, Zhang Y, Will R, Kenyon RW, Sobral BW. 2011. PATRIC: the Comprehensive Bacterial Bioinformatics Resource with a Focus on Human Pathogenic Species ▿. Infect Immun 79:4286–4298.

39. Tatusova T, Ciufo S, Fedorov B, O’Neill K, Tolstoy I. 2014. RefSeq microbial genomes database: new representation and annotation strategy. Nucleic Acids Res 42:D553–D559.

40. Karp PD, Billington R, Caspi R, Fulcher CA, Latendresse M, Kothari A, Keseler IM, Krummenacker M, Midford PE, Ong Q, Ong WK, Paley SM, Subhraveti P. 2017. The BioCyc collection of microbial genomes and metabolic pathways. Brief Bioinform 28:1–6.

41. Kanehisa M, Furumichi M, Tanabe M, Sato Y, Morishima K. KEGG: new perspectives on genomes, pathways, diseases and drugs. Nucleic Acids Res 45.

42. Newton GL, Leung SS, Wakabayashi JI, Rawat M, Fahey RC. 2011. The DinB Superfamily Includes Novel Mycothiol, Bacillithiol, and Glutathione S-Transferases. Biochemistry 50:10751–10760.

43. Singhal N, Sharma P, Kumar M, Joshi B, Bisht D. 2012. Analysis of intracellular expressed proteins of Mycobacterium tuberculosis clinical isolates. Proteome Sci 10:1–10.

44. Wang C, Zhang Q, Wang Y, Tang X, An Y, Li S, Xu H, Li Y, Luan W, Wang X, Liu M, Yu L. 2019. Comparative proteomics analysis between biofilm and planktonic cells of *Mycobacterium tuberculosis*. Electrophoresis 40:2736–2746.

45. Gao C, Yang M, He Z-G. 2012. Characterization of a Novel ArsR-Like Regulator Encoded by Rv2034 in Mycobacterium tuberculosis. PLoS One 7:e36255.

46. Gao CH, Yang M, He ZG. 2011. An ArsR-like transcriptional factor recognizes a conserved sequence motif and positively regulates the expression of phoP in mycobacteria. Biochem Biophys Res Commun 411:726–731.

47. Boradia VM, Malhotra H, Thakkar JS, Tillu VA, Vuppala B, Patil P, Sheokand N, Sharma P, Chauhan AS, Raje M, Raje CI. 2014. Mycobacterium tuberculosis acquires iron by cell-surface sequestration and internalization of human holo-transferrin. Nat Commun 5:1–13.

48. Baena A, Cabarcas F, Alvarez-Eraso KLF, Isaza JP, Alzate JF, Barrera LF. 2019. Differential determinants of virulence in two Mycobacterium tuberculosis Colombian clinical isolates of the LAM09 family. Virulence 10:695–710.

49. Gandotra S, Schnappinger D, Monteleone M, Hillen W, Ehrt S. 2007. In vivo gene silencing identifies the Mycobacterium tuberculosis proteasome as essential for persistence in mice. Nat Med 13:1515–1520.
