# Supplementary figures and images for: Structure-Aware Mycobacterium tuberculosis Functional Annotation Uncloaks Resistance, Metabolic, and Virulence Genes
Source: mSystems. 2021 Nov 2;6(6):e00673-21. doi: 10.1128/mSystems.00673-21 (PMC8562490; doi:10.1128/mSystems.00673-21)

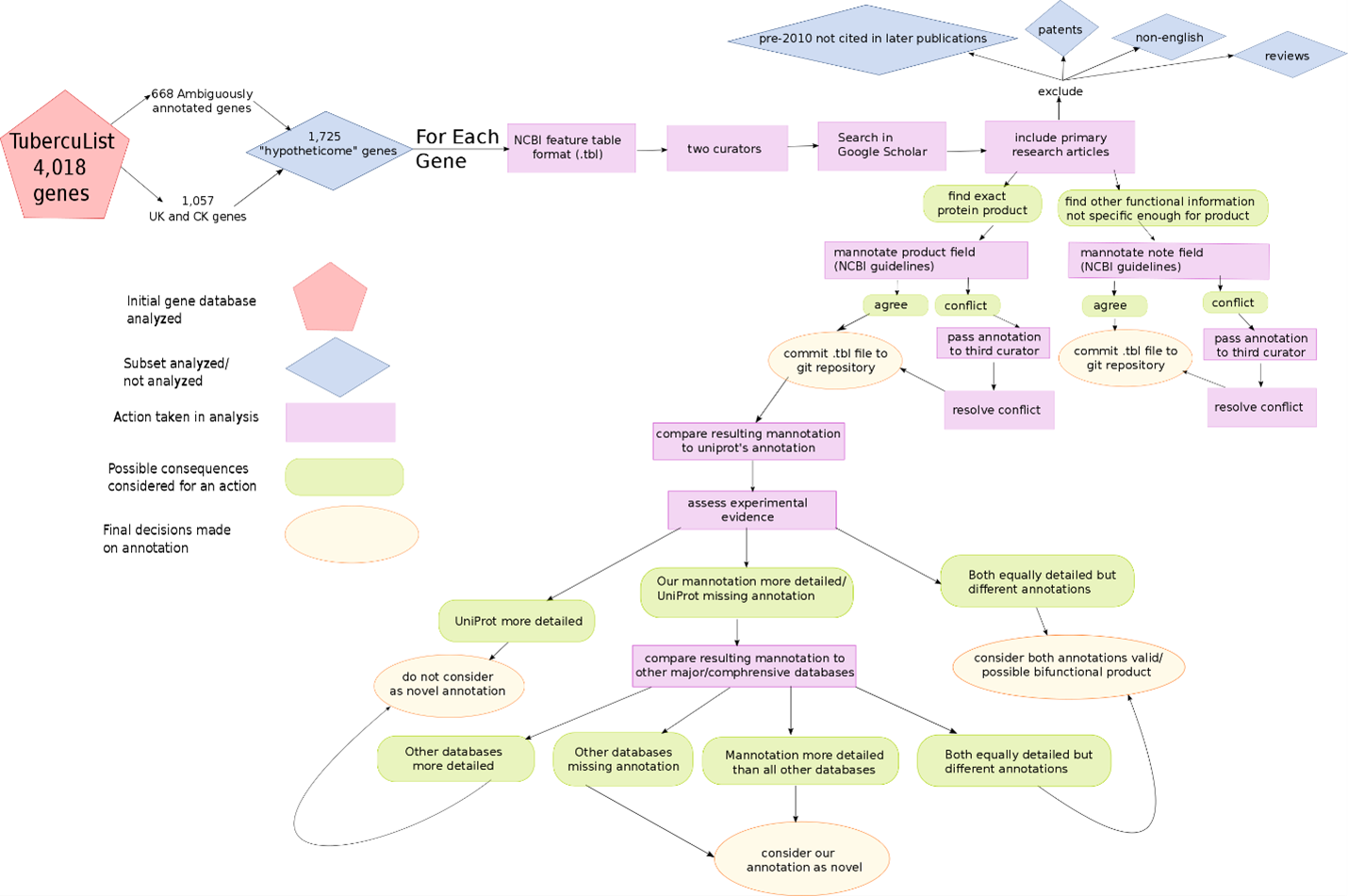

Supplement: FIG S1 [file msystems.00673-21-sf001.tif]

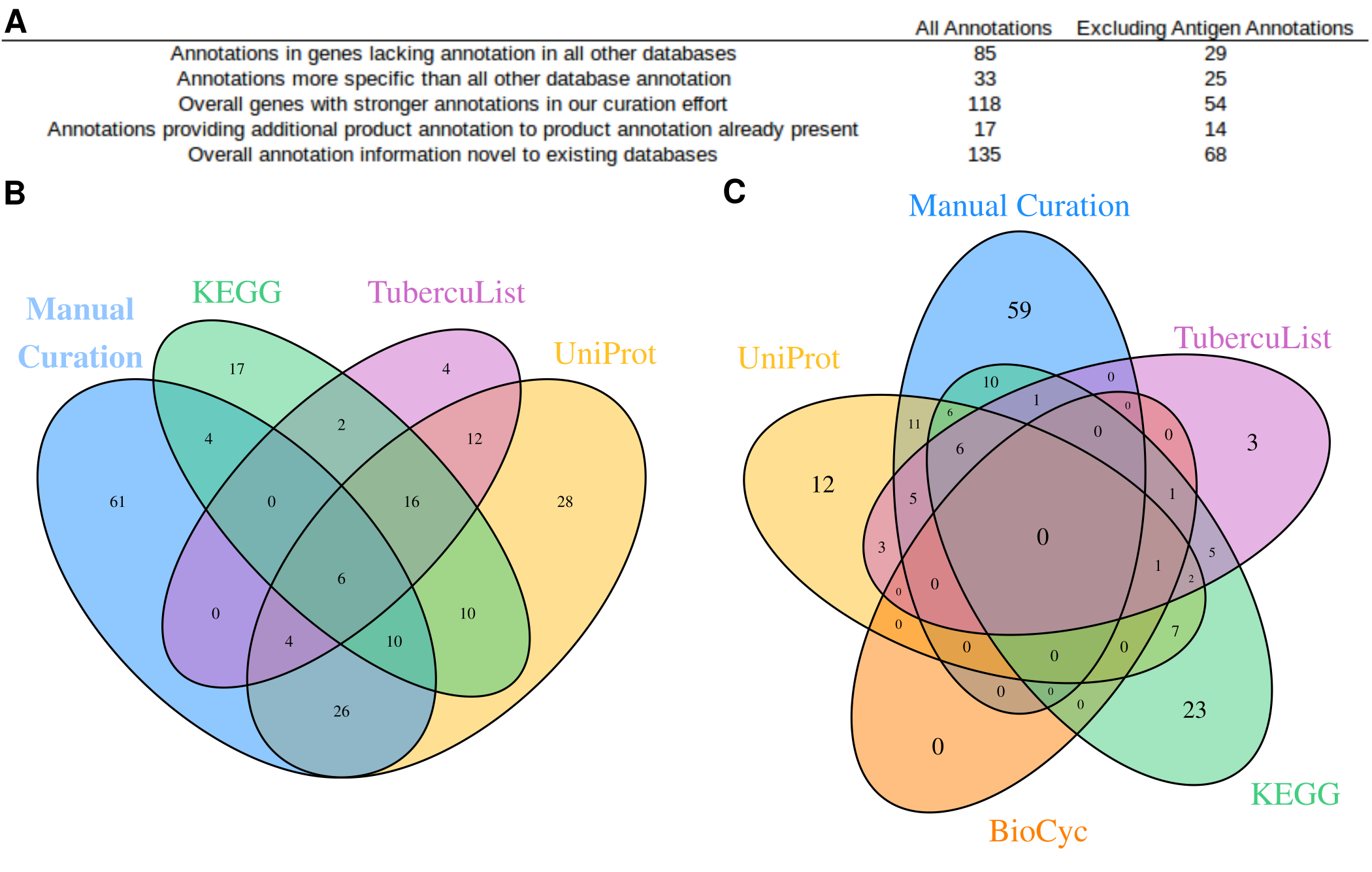

Supplement: FIG S4 [file msystems.00673-21-sf004.tif]

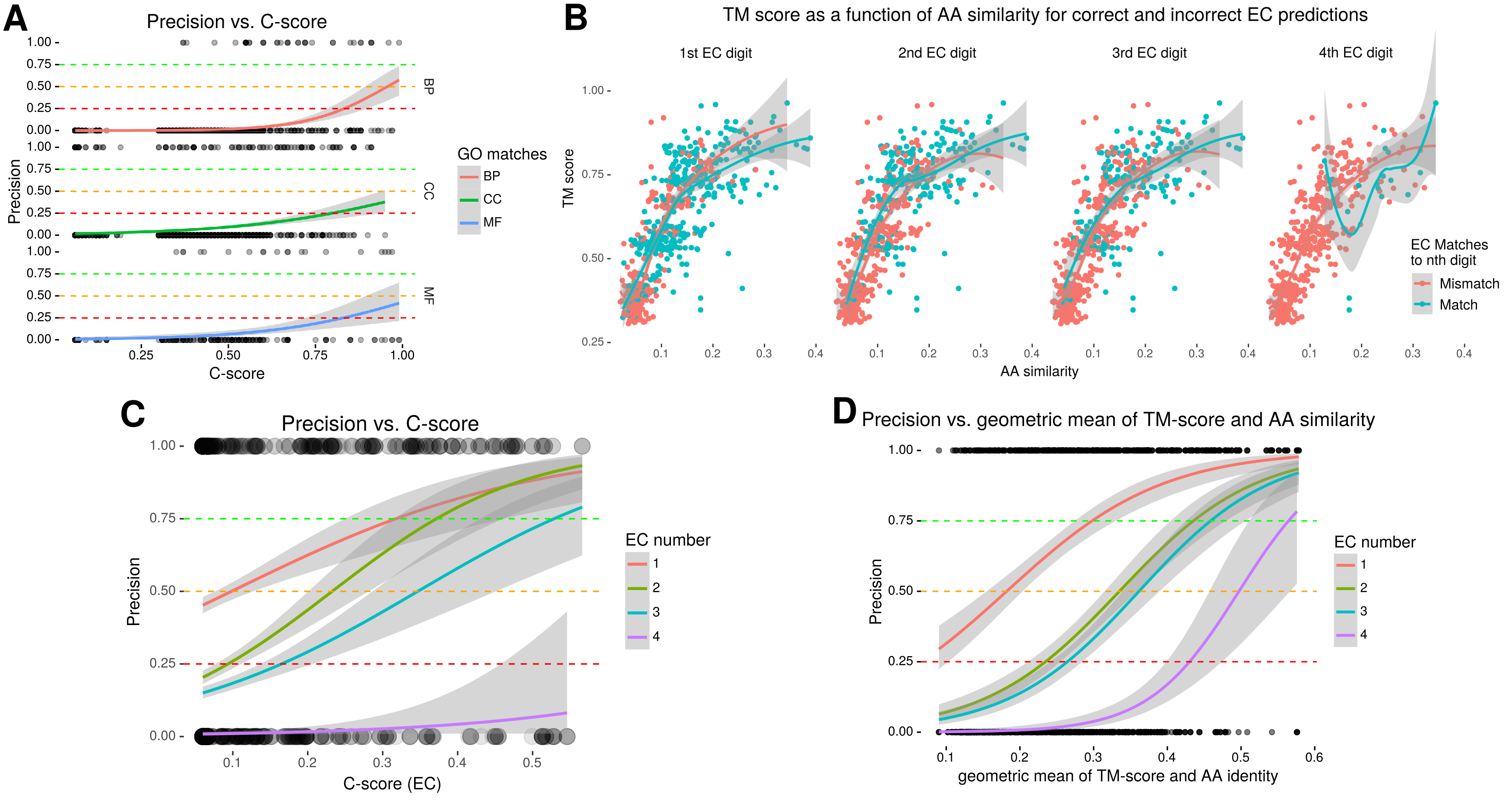

Supplement: FIG S2 [file msystems.00673-21-sf002.tif]

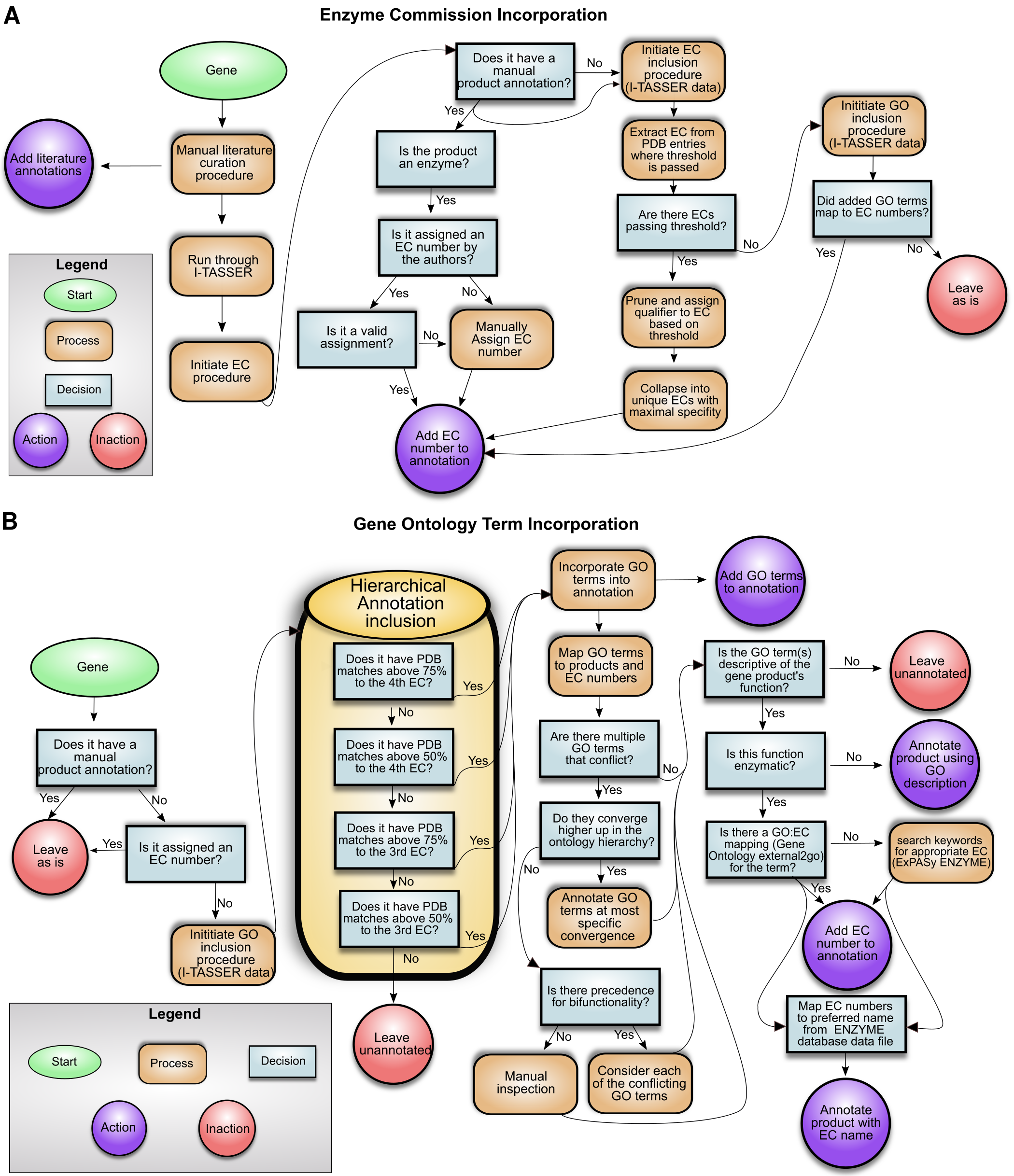

Supplement: FIG S3 [file msystems.00673-21-sf003.tif]

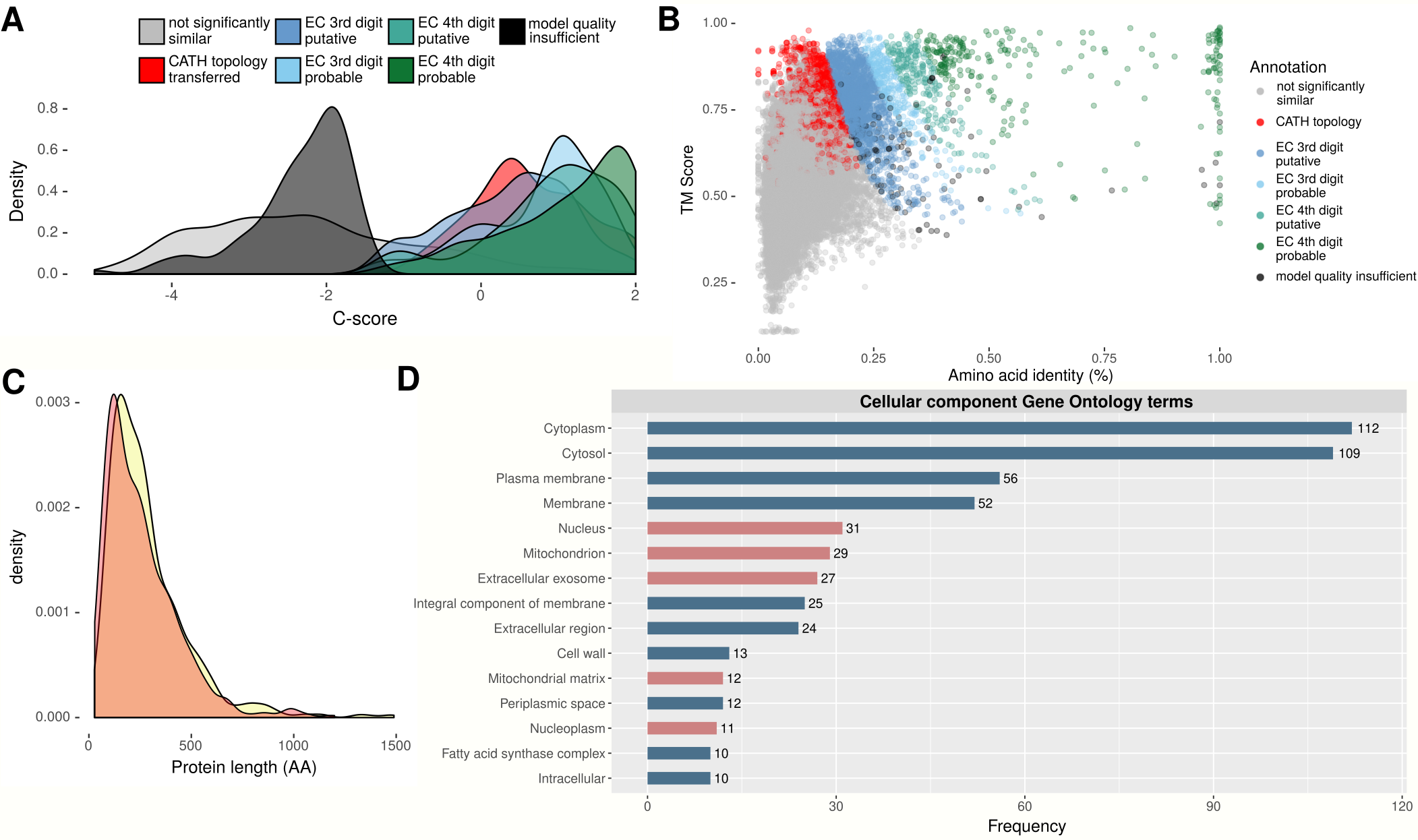

Supplement: FIG S5 [file msystems.00673-21-sf005.tif]
